# Secure Base Leadership, Organizational Dehumanization, and Psychological Safety Among Intensive Care Nurses: A Cross‐Sectional Study

**DOI:** 10.1111/inr.70232

**Published:** 2026-09-04

**Authors:** Susana Montenegro‐Méndez, Juan A. Moriano León, Ana Laguía González

**Affiliations:** ^1^ Psychology Program, Escuela Internacional de Doctorado de la Universidad Nacional de Educación a Distancia (EIDUNED) Madrid Spain; ^2^ Department of Social and Organizational Psychology Universidad Nacional de Educación a Distancia (UNED) Madrid Spain

**Keywords:** burnout, intensive care nurses, organizational dehumanization, psychological safety, secure base leadership, work engagement

## Abstract

**Aim:**

Intensive care unit (ICU) nurses operate in highly demanding environments. Secure base leadership (SBL) has been proposed as a resource to help manage these demands. This study examines the associations between ICU nurses’ perceptions of their supervisor as a secure base leader and their burnout and work engagement (WE), as well as the pattern of indirect statistical associations involving organizational dehumanization (OD) and psychological safety (PS) between SBL and these outcomes.

**Design:**

A cross‐sectional study conducted in accordance with the Strengthening the Reporting of Observational Studies in Epidemiology (STROBE) guidelines.

**Methods:**

A total of 305 ICU nurses from Spain completed an online questionnaire comprising established measures assessing SBL, OD, PS, burnout, WE, and sociodemographic variables. Data were analyzed using partial least squares structural equation modeling (PLS‐SEM).

**Results:**

The direct associations between SBL and burnout and WE were not statistically significant. SBL was significantly associated with lower OD and higher PS, and OD and PS were significantly associated with lower burnout and higher WE. The final model explained 20% of the variance in burnout and 15% of the variance in WE.

**Conclusion:**

SBL was associated with ICU nurses’ burnout and WE in a pattern consistent with indirect statistical associations involving OD and PS, whereas the direct associations between SBL and burnout and WE were not statistically significant. Given the cross‐sectional design, these findings should not be interpreted as evidence of causal or temporal mediation.

**Implications for Nursing:**

In highly demanding clinical settings, SBL may represent a relevant leadership resource in relation to more humanized and psychologically safe work environments.

**Implications for Nursing Policy:**

Nursing leadership development strategies may benefit from prioritizing PS and addressing OD. Such approaches are consistent with the factors associated with nurses’ well‐being and engagement in high‐pressure healthcare settings.

## 1 Introduction

Intensive care units (ICUs) represent one of the most challenging environments in healthcare, where nurses face extraordinary physical, emotional, and psychological demands. The nature of the work requires nurses to maintain constant vigilance and provide high‐intensity interventions (Perveen 2022; Yin et al. 2024).

Drawing on a systematic review that underscored the importance of leadership in managing burnout and promoting work engagement (WE) in nursing (Montenegro Méndez et al. 2025), previous research supports theoretical models such as the Job Demands–Resources Model (JD‐R; Bakker et al. 2023). Within this framework, job resources (e.g., leadership, organizational support, and positive work environment) can mitigate job demands (e.g., emotional stress, workload), thereby promoting WE and preventing burnout (Padilha et al. 2017; Ramírez‐Elvira et al. 2021).

Previous studies have examined various leadership styles in nursing, highlighting their associations with care practices, staff attitudes, and well‐being (Montenegro Méndez et al. 2025). Among these, secure base leadership (SBL) has recently emerged as a relational and caring approach that is gaining increasing empirical attention across different sectors (Laguia et al. 2025; Lobato et al. 2024; Moriano et al. 2021; Navas‐Jiménez et al. 2024, 2025). However, SBL has yet to be specifically investigated in ICU nursing. SBL has been proposed as a promising leadership framework in this setting, with growing evidence suggesting that it is positively associated with staff well‐being, quality of care, performance, and ethical conduct (Movaghar et al. 2024).

Organizational dehumanization (OD) and psychological safety (PS) are considered critical factors in nursing, being directly associated with healthcare quality, professional well‐being, and patient care (Cimon et al. 2024). In ICUs, OD correlates with lower care quality and higher burnout risk (Iacopo et al. 2024), while PS is positively linked to nurses' mental health when facing constant high‐pressure situations and critical decisions, making it essential for maintaining effective and sustainable care (Intriago Moreira et al. 2025).

### 1.1 Secure Base Leadership (SBL)

Grounded in Bowlby's (1982) attachment theory, SBL is a relational approach where leaders, through their proximity, establish positive emotional bonds by providing protection, security, and care, especially in times of stress or uncertainty. At the same time, SBL provides a foundation of trust that inspires and energizes team members to dare to explore, take risks, and face new challenges with initiative and confidence (Molero et al. 2019; Moriano León 2025).

SBL represents a paradigm shift from “command and control” to relational and collaborative leadership. This is particularly relevant in ICU nursing, where care requires high emotional intelligence and interpersonal connection (de Zulueta 2015).

### 1.2 Burnout

Burnout, defined as a psychological syndrome involving emotional exhaustion, depersonalization, and reduced personal achievement (Maslach and Jackson 1981), remains highly prevalent among ICU nurses, with rates between 17% and 61% (Ramírez‐Elvira et al. 2021). Factors such as anxiety, stress, and alarm fatigue are critical drivers of burnout in ICU nurses (Yousif and Al‐Fayyadh 2025). Consequently, ICU supervisors may play a crucial role in fostering supportive work environments, which have been associated with lower levels of burnout (Epp 2012). Based on this, we propose the following hypothesis:

Hypothesis 1a SBL will be negatively related to job burnout among ICU nurses.

### 1.3 Work Engagement (WE)

Initially, WE was viewed as the opposite of burnout (Maslach and Leiter 2008). However, the JD‐R model (Bakker et al. 2023; Demerouti et al. 2001) offered a more comprehensive framework, establishing them as distinct yet related phenomena influenced by the balance between job demands and resources. Schaufeli et al. (2002) define WE as a positive mental state toward work, characterized by vigor, dedication, and absorption.

Studies in ICUs report moderate to high WE levels, suggesting that nurses maintain engagement despite the high‐stress environments, though variability exists depending on workload, personal qualifications, and organizational factors (Haruna et al. 2023). Therefore, we formulate the following hypothesis:

Hypothesis 1b SBL will be positively related to WE among ICU nurses.

### 1.4 Organizational Dehumanization (OD)

Dehumanization in organizations occurs when employees are treated as mere objects or resources, stripped of their emotions and human characteristics (Haslam 2006). This instrumental approach views individuals solely as means to an end, ignoring their dignity and creating toxic, alienating environments (Moriano León 2025).

Caring for people is the antidote to OD (Moriano León 2025), and leadership plays an essential role in this process, as it is the primary vehicle for promoting relationships based on empathy, trust, and recognition. Indeed, SBL has been negatively associated with dehumanization in both military (Lobato et al. 2024) and civilian (Moriano et al. 2021) samples. We therefore posit the following hypothesis:

Hypothesis 2 SBL will be negatively related to OD.

Extensive research indicates that OD is positively related to burnout (Wang et al. 2023) and negatively related to WE (Abou Zeid et al. 2024) among nurses. In particular, SBL is associated with lower levels of OD, which has consistently been associated with higher burnout and stress (Lobato et al. 2024; Moriano et al. 2021) and lower WE (Laguia et al. 2025; Molero et al. 2019; Navas‐Jiménez et al. 2024). Consequently, the following hypotheses are formulated:

Hypothesis 3a OD will be positively related to burnout.

Hypothesis 3b OD will be negatively related to WE.

### 1.5 Psychological Safety (PS)

A secure base leader not only provides guidance and support when the team faces uncertainties or problems, but also creates an environment of PS (Edmondson 1999). This approach facilitates well‐being, allowing team members to manage stress more effectively by trusting that they can rely on their leader in challenging situations (Moriano León 2025).

PS is a fundamental construct in ICUs, where nurses face high‐pressure environments, critical decisions, and emotionally challenging situations. Defined as a climate safe for interpersonal risk‐taking, PS has emerged as a crucial factor for nursing staff well‐being and patient care quality in these settings (Grailey et al. 2021).

Previous studies have found that leadership styles encouraging the participation of nurses and residents are negatively correlated with the perception of futile care and positively correlated with the team's PS (Schwarzkopf et al. 2015). Consistently, SBL has been positively associated with a PS climate, fostering an environment where employees feel safe to express themselves and take interpersonal risks without fear of negative consequences (Lobato et al. 2024; Moriano et al. 2021). This leads to the formulation of the following hypothesis:

Hypothesis 4 SBL will be positively related to PS.

Moreover, evidence suggests that PS reduces burnout by mitigating job‐related stress and protecting employees from emotional exhaustion and disengagement (Edmondson and Lei 2014). Since PS in nursing teams varies according to the organizational context (Grailey et al. 2021), we formulate the following hypotheses:

Hypothesis 5a PS will be negatively related to burnout.

Hypothesis 5b PS will be positively related to WE.

**FIGURE 1:**
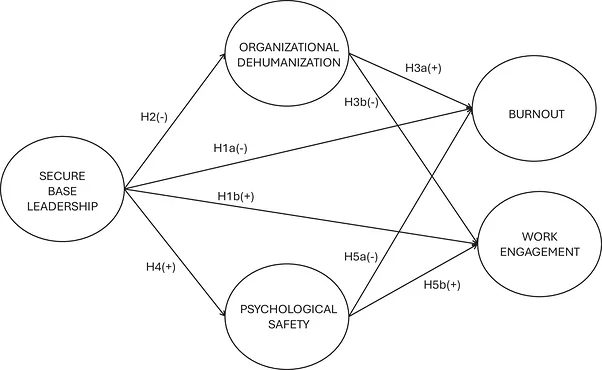
Theoretical model and hypotheses.

The conceptual model and derived hypotheses are illustrated in Figure 1, depicting a pattern of associations in which SBL is negatively related to burnout and positively related to WE among ICU nurses, with PS and OD specified as intermediary variables in the proposed statistical model.

### 1.6 Aim

The primary aim of this study is twofold. First, to examine the associations between perceived SBL and self‐reported burnout and WE among ICU nurses. Second, to explore patterns of indirect statistical associations involving OD and PS within the proposed theoretical model.

## 2 Methods

### 2.1 Design, Procedure, and Participants

A cross‐sectional, correlational design was employed. ICU nurses in Spain were invited to participate through professional associations, mailing lists, and social media. The questionnaire was administered via Qualtrics, ensuring confidentiality and anonymity. Since the link was shareable, the total number of individuals who received the invitation is unknown, and the formal response rate could not be determined. Data were collected between April 2025 and September 2025. All participants completed the scales in the same order of presentation.

Eligibility criteria were being a registered nurse currently working in an ICU and voluntarily agreeing to participate by providing informed consent. Nursing students were excluded. A total of 467 questionnaires were received. The analyses were conducted using a complete‐case approach. Questionnaires that were not completed in full were excluded, and no imputation of missing data was performed. The final sample therefore consisted of all eligible participants who provided complete responses to all study variables. After excluding cases with missing data, 305 responses were included in the final analysis (questionnaire completion rate: 65.3%).

The sample was predominantly female (80.7%), with the largest age groups being 36–45 (31.5%) and 46–55 (28.9%). Most participants were highly experienced: 74.1% had over 10 years of practice, and 39.8% had worked with their current supervisor for 1–5 years. Regarding the work context, 54.8% were employed in public healthcare centers, 34.8% in private institutions, and 75.3% worked in adult ICUs. Work schedules varied, with 25.6% reporting rotating shifts and 32.8% working standard daytime hours. In terms of workload, nearly half of the participants (49.5%) reported caring for two patients per shift.

### 2.2 Measures

#### 2.2.1 Secure Base Leadership

To measure ICU nurses’ perceptions of their supervisor as a secure base leader, we used the Leader as Security Provider Scale (LSPS; Molero et al. 2019) consisting of 15 original items supplemented by 5 additional items to enhance the scale's robustness. This unidimensional scale assesses the four fundamental characteristics of attachment figures described in the theoretical framework: secure base, safe haven, proximity seeking, and emotional bond. All items were formulated to focus participants’ attention on their direct supervisor (e.g., “I can count on my leader to support me when I propose new ideas or procedures”). Participants indicated their level of agreement using a five‐point Likert scale ranging from 0 (*not agree at all*) to 4 (*totally agree*). Scores were computed as the mean of the 20 items, with higher scores indicating a stronger perception of the supervisor as a secure base leader.

#### 2.2.2 Burnout

Burnout was assessed using the Spanish version validated by Soriano et al. (2025) of the Burnout Assessment Tool (BAT; Schaufeli et al. 2020). This 12‐item instrument assesses the extent to which individuals experience burnout in their current work situation across four dimensions: exhaustion, emotional impairment, cognitive impairment, and mental distance (e.g., “At work, I feel mentally exhausted”). Participants rated each item on a five‐point Likert scale ranging from 0 (*never*) to 4 (*every day*). Scores were computed as the mean across dimensions, with higher scores indicating higher levels of burnout.

#### 2.2.3 Work Engagement

WE was measured using the Spanish version of the UWES‐3 (Schaufeli et al. 2019). This brief scale includes one item for each of the core dimensions of WE: vigor, dedication, and absorption (e.g., “At my work, I feel bursting with energy”). Participants rated all items on a five‐point Likert scale ranging from 0 (*never*) to 4 (*every day*). Scores were computed as the mean of the three items, with higher scores indicating higher levels of WE.

#### 2.2.4 Organizational Dehumanization

OD was measured using the 11‐item scale developed by Caesens et al. (2017), validated in Spanish by Ariño‐Mateo et al. (2022). This instrument assesses the extent to which employees perceive that their organization treats them as interchangeable resources rather than as individuals (e.g., “My organization considers me as a mere tool to achieve its goals”). Item 1 (“My organization makes me feel that a worker is easily as good as any other”) was removed due to poor psychometric properties (loading = −.17), consistent with previous findings (this item may be ambiguously interpreted, as it can be perceived as either a positive or negative organizational characteristic). Its exclusion improved internal consistency (α increased from .93 to .95) and convergent validity (average variance extracted (AVE) from .65 to .71), following established recommendations for scale refinement (DeVellis and Thorpe 2021; Hair et al. 2019). Participants rated all items on a five‐point Likert scale ranging from 0 (*not agree at all*) to 4 (*totally agree*). Scores were computed as the mean of the 10 items, with higher scores indicating higher levels of perceived OD.

#### 2.2.5 Psychological Safety

PS was measured with Edmondson's (1999) 7‐item scale, validated in Spanish by Fajardo Castro et al. (2024). This instrument assesses the extent to which individuals perceive their team as a safe environment for interpersonal risk‐taking (e.g., “It is safe to take a risk on this team”). For the analysis, item 6 (“No one on this team would deliberately act in a way that would undermine my efforts”) was removed due to a negative loading (−.28). Previous research has shown that this item may be problematic due to its wording, as respondents tend to interpret it as negatively formulated, which can affect its psychometric performance (Baer and Frese 2003). This refinement improved the scale's reliability (α from .65 to .76) and validity (AVE from .39 to .45), following established recommendations for scale refinement (DeVellis and Thorpe 2021; Hair et al. 2019). Participants rated all items on a five‐point Likert scale ranging from 0 (*never*) to 4 (*always*). Scores were computed as the mean of the six items, with higher scores indicating higher levels of PS.

#### 2.2.6 Sociodemographic Variables

Participants reported sociodemographic information (e.g., gender and age), professional characteristics (e.g., years of experience and tenure with their current supervisor), and contextual data related to their work setting, including type of healthcare center, work shift, type of ICU, and number of patients cared for per shift.

### 2.3 Data Analysis

Descriptive statistics were calculated using Jamovi 2.6.44 (Jamovi Project 2024). The measurement and structural models were estimated via partial least squares structural equation modeling (PLS‐SEM) using SmartPLS (Ringle et al. 2022). PLS‐SEM was selected for its suitability for analyzing complex models with multiple mediators and its robustness with nonnormal data (Hair et al. 2022). The analysis followed a two‐step approach, assessing first the measurement model and subsequently the structural model. Path significance was evaluated using bootstrapping with 5000 resamples. Multicollinearity was ruled out as all variance inflation factor values were below commonly accepted thresholds, indicating no collinearity issues. Consistent with the study's focus on the relationships between focal psychological constructs, sociodemographic variables were used solely for descriptive purposes and were not included in the structural model.

No formal a priori sample size calculation was performed because convenience sampling was used, and all eligible questionnaires completed during the study period were included in the analyses. To evaluate the adequacy of the final sample for the proposed PLS‐SEM model, a post hoc assessment was conducted using the inverse square root method implemented in SmartPLS (Kock and Hadaya 2018). The minimum required sample size varied according to the magnitude of the structural path coefficients. For the statistically significant structural relationships, the required sample sizes ranged from 57 to 286 participants (α = .05; power = 90%), all of which were exceeded by the final sample of 305 nurses. The only paths requiring substantially larger samples corresponded to very small direct associations between secure base leadership and burnout and WE, suggesting that very small direct associations may have remained undetected.

## 3 Results

To assess the potential impact of common method bias (CMB), Harman's single‐factor test was conducted using exploratory factor analysis with maximum likelihood estimation (Harman 1976; Podsakoff et al. 2003). The results did not indicate the presence of a dominant single factor, with the first unrotated factor accounting for 31.6% of the total variance.

Spearman correlations revealed that perceptions of SBL were significantly associated with higher levels of PS and WE, and lower levels of OD and burnout (Table 1). Additionally, in line with our hypotheses, burnout was negatively associated with PS and positively with OD, while WE was positively associated with PS and negatively with OD.

**TABLE 1:** Descriptive statistics, reliability, validity, and correlations among study variables.

Constructs	Mean	SD	AVE	CR	α	1	2	3	4	5
SBL	2.62	1.04	.72	.98	.98					
Burnout	1.39	0.62	.57	.76	.76	−.17^**^				
WE	2.60	0.90	.70	.80	.79	.25^***^	−.33^***^			
OD	2.60	1.12	.71	.95	.95	−.33^***^	.35^***^	−.28^***^		
PS	2.46	0.75	.45	.77	.76	.39^***^	−.36^***^	.32^***^	−.41^***^	

*Note*: *N*, 305. SBL, Secure Base Leadership. WE, Work Engagement. OD, Organizational Dehumanization. PS, Psychological Safety. AVE, Average Variance Extracted. CR, Composite Reliability.

** *p* < .01. ^***^ *p* < .001.

### 3.1 Measurement Models

Individual item reliability was confirmed as all standardized factor loadings (λ) of each latent construct were statistically significant (*p* < .001). All factor loadings exceeded .60, except for item 2 of the PS scale (λ = .52). Internal consistency was supported by Cronbach's alpha (α) and composite reliability (CR) values (Table 1).

Convergent validity was measured through the AVE, which reflects the total amount of variance of the indicators collected by the latent variable. All constructs exceeded the .50 cutoff value (Table 1), except for PS (AVE = .45). Discriminant validity was established using the heterotrait–monotrait (HTMT) ratio (Hair et al. 2022); all ratios were below the conservative .85 threshold (the highest value corresponded to HTMT_burnout‐PS_ = .45).

### 3.2 Structural Model

**FIGURE 2:**
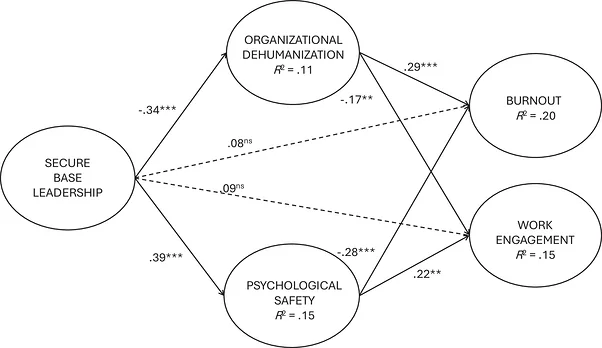
Standardized regression coefficients for the full structural model. Note. ns = nonsignificant. ** *p* < .01. *** *p* < .001. Solid lines indicate statistically significant paths, whereas dashed lines indicate nonsignificant paths.

The direct path model did not provide support for H1a or H1b, as the hypothesized paths from SBL to burnout (ß = .08, *p* = .17) and to WE (ß = .09, *p* = .09) were not statistically significant. However, the full structural model (Figure 2) showed that SBL was negatively associated with OD (ß = −.34, *p* < .001) and positively with PS (ß = .39, *p* < .001), supporting H2 and H4. Moreover, OD and PS were significantly associated with burnout (ß = .29, *p* < .001 and ß = −.28, *p* < .001, respectively) and WE (ß = −.17, *p* = .003 and ß = .22, *p* = .002, respectively), thereby supporting H3a, H3b, H5a, and H5b. The coefficients of determination for OD (*R* ^2^ = .11) and PS (*R* ^2^ = .15) exceeded the minimum recommended value (.10). In this full structural model, the total associations between SBL and both burnout and WE were significant (ß = −.20, *p* < .001 and ß = .14, *p* < .001, respectively). These findings were consistent with a pattern of indirect statistical associations involving OD and PS in the associations between SBL and the outcomes. Table 2 summarizes the direct and indirect associations and their statistical significance.

**TABLE 2:** Structural model: Direct, indirect, and total associations.

Associations	β	95% CI (Percentile)	95% CI (BC)
Direct paths			
H1a: SBL—Burnout	.08	[−.03, .21]	[−.04, .21]
H1b: SBL—WE	.09	[−.02, .20]	[−.01, .21]
H2: SBL—OD	−.34	[−.42, −.25]	[−.42, −.24]
H3a: OD—Burnout	.29	[.19, .39]	[.18, .38]
H3b: OD—WE	−.17	[−.28, −.05]	[−.28, −.05]
H4: SBL—PS	.39	[.27, .50]	[.26, .49]
H5a: PS—Burnout	−.28	[−.40, −.16]	[−.38, −.14]
H5b: PS—WE	.22	[.08, .37]	[.07, .36]
Indirect paths			
H2 x H3a: SBL—OD—Burnout	−.10	[−.14, −.06]	[−.14, −.06]
H2 x H3b: SBL—OD—WE	.06	[.01, .10]	[.01, .10]
H4 x H5a: SBL—PS—Burnout	−.10	[−.17, −.06]	[−.16, −.05]
H4 x H5b: SBL—PS—WE	.08	[.03, .16]	[.03, .16]
Total indirect associations			
H1a: SBL—Burnout	−.20	[−.28, −.15]	[−.27, −.14]
H1b: SBL—WE	.14	[.08, .22]	[.08, .21]

*Note*: SBL, Secure Base Leadership. WE, Work Engagement. OD, Organizational Dehumanization. PS, Psychological Safety. Associations are significant when the 95% confidence interval does not include zero.

The model explained 20% of the variance in burnout and 15% in WE. Finally, all *Q* ^2^ predicted values for endogenous variables were greater than zero, confirming the model's explanatory relevance.

## 4 Discussion

Preliminary analysis showed that SBL was significantly correlated with both burnout (H1a) and WE (H1b). However, these direct associations between SBL and these outcomes were not statistically significant in the structural model, whereas OD and PS remained significantly associated with burnout and WE. Taken together, these findings indicate a pattern of indirect statistical associations involving OD and PS that is consistent with the proposed theoretical model. Accordingly, OD and PS may be considered relevant correlates of nurse well‐being within the proposed model.

From a theoretical perspective, this underlines a distinction between SBL and other leadership approaches. Previous research has consistently shown that transformational leadership is directly associated with WE and burnout in ICU nurses. For example, recent studies have reported significant direct relationships between transformational leadership and WE, as well as indirect associations through organizational climate (Zhang et al. 2025), and both direct and indirect associations with burnout and related outcomes in intensive care settings (Guo et al. 2022). In contrast, SBL, as conceptualized in this study, emphasizes the provision of a secure relational base through support, availability, and humane treatment. Rather than showing direct statistical associations with burnout or WE, the observed pattern was consistent with indirect statistical associations involving SBL, OD, PS, and both outcomes, as proposed in the conceptual model. These findings suggest that the associations between SBL and employee well‐being outcomes may be related to relational and organizational conditions in the workplace. In this sense, OD and PS were significantly associated with both burnout and WE within the proposed model and formed part of the pattern of indirect statistical associations involving SBL and these outcomes. This interpretation is also consistent with previous research reporting positive associations between leadership characterized by emotional support and trust and employee engagement and authentic contribution (Konečná et al. 2026).

As hypothesized (H2), SBL was negatively related to OD. Aligning with Moriano et al. (2021) and Lobato et al. (2024), being cared and treated fairly and personally by a secure base leader may be relevant to lower perceptions of OD and its associated psychological and organizational consequences. As expected, OD was positively associated with burnout and negatively with WE. Healthcare organizations may consider the relevance of leaders fostering humanized work environments to improve nurses’ work engagement and reduce their burnout. Future research should examine whether such leadership is also associated with patient care and ICU nurse retention (Quesada‐Puga et al., 2024). Similar to other studies (Cimon et al. 2024; Iacopo et al. 2024), our results also show the high average scores for feelings of dehumanization, suggesting that many ICU nurses feel treated as mere objects of production, experiencing organizational coldness, indifference, and invisibility.

Furthermore, SBL was positively related to PS (H4). By caring for subordinates’ safety and welfare and supporting and encouraging their performance, secure base leaders tend to be associated with a PS climate where team members feel safe to disclose and learn from mistakes without fear of retribution (Lobato et al. 2024; Moriano et al. 2021). Although nonpunitive climates and support programs are encouraged in ICUs (Kappes et al. 2023), the average PS scores obtained in this study indicate that healthcare organizations may consider implementing support systems that demonstrate that they care about the well‐being of nurses, value their opinions, and take pride in their achievements (Saravanabavan and Poongavanam 2023).

### 4.1 Limitations

These findings should be interpreted considering several limitations. First, men were underrepresented, though this distribution is consistent with the Spanish nursing workforce (84.20% female; INE 2024). Second, the cross‐sectional design precludes causal inferences; while theoretically grounded, the directionality of the associations cannot be empirically established. Third, the use of a convenience sample drawn from different healthcare organizations may limit the generalizability of the findings. Although all eligible participants who completed the questionnaire during the study period were included, the sample size may have provided limited sensitivity to detect very small direct associations within the structural model. Consequently, the absence of statistically significant direct associations should be interpreted with caution. The exclusion of incomplete questionnaires may also have introduced bias, as potential differences between completers and noncompleters could not be assessed due to the lack of demographic information for noncompleters. Fourth, all variables were collected through self‐report in the same survey; although Harman's single‐factor test did not indicate a dominant factor, this approach cannot completely rule out the possibility of CMB. Nevertheless, the instruments employed are validated in Spanish samples and have demonstrated adequate psychometric properties. Fifth, several items were removed during scale refinement. While the remaining items showed adequate reliability, this may have narrowed the conceptual domain of certain constructs. Future research should further examine these reduced measures across diverse cultural and organizational contexts. Sixth, PS showed suboptimal convergent validity (AVE < .50), although its composite reliability was acceptable. This suggests that the indicators may not have captured the construct as robustly as expected, potentially leading to conservative estimates of its associations. Future studies should refine the measurement of PS in ICU settings, for example, by revising existing items or incorporating additional indicators that better reflect this construct in highly demanding clinical environments. Finally, as data reflect ICU nurses’ perceptions of their supervisors as secure base leaders, future research should incorporate multiple sources (e.g., supervisors’ self‐reports) and longitudinal or multilevel designs.

### 4.2 Implications for Nursing and Health Policy

Health policies may consider SBL training programs for ICU supervisors as a potential component of leadership development initiatives aimed at supporting PS and addressing OD. The observed associations between SBL, PS, OD, burnout, and WE suggest that SBL training initiatives may be relevant for supporting nurse well‐being and engagement in the context of ongoing healthcare transformations, including digitalization. International nursing organizations may also consider advocating for regulatory frameworks ensuring humanized work environments, ICU workforce sustainability, and global patient safety. However, SBL training was not evaluated in the present study; therefore, the potential effectiveness of interventions aimed at developing SBL behaviors should be examined in future longitudinal and intervention studies.

## 5 Conclusion

SBL may represent a relevant leadership resource for ICU nurses. The observed pattern of associations suggests that the relationship between leadership and nurses’ well‐being is more closely linked to psychologically safe and humanized work environments rather than to direct associations alone. In a context of profound transformation, these findings suggest that SBL may be relevant to supporting ICU nurses’ well‐being. Further research, particularly longitudinal and intervention studies, is needed to evaluate whether strategies aimed at developing SBL behaviors can contribute to more psychologically safe, humanized, and supportive work environments in these highly demanding settings.

## Author Contributions

**Susana Montenegro Méndez**: conceptualization, methodology, formal analysis, writing – original draft, writing – review and editing. **Juan A. Moriano León**: conceptualization, resources, methodology, writing – original draft. **Ana Laguía González**: conceptualization, methodology, data curation, writing – original draft, writing – review and editing.

## Funding

The authors have nothing to report.

## Ethics Statement

The Ethics Committee of the National University of Distance Education (UNED, Spain), in accordance with the suitability requirements established by this Committee for projects involving research with human subjects, issued a favorable report on the project (Reference: 27‐SISH‐PSI‐2025).

## Conflicts of Interest

No potential conflict of interest was reported by the authors.

## Use of Artificial Intelligence

ChatGPT 5.0o (OpenAI) was utilized for language editing, grammar correction, and translation to ensure clarity and accuracy in the English version of the text. No AI tools were used for data analysis, content generation, or research interpretation.

## Data Availability

The data that support the findings of this study are available from the corresponding author upon reasonable request.

## References

[inr70232-bib-0001] Abou Zeid, M.‐A. G. , M. A. Khedr , H. N. Rayan , B. Mostafa , and A. M. El‐Ashry . 2024. “The Relationship Between Organizational Dehumanization and Work Engagement: The Mediating Effect of Nurses' Work Stress.” BMC Nursing 23, no. 1: 193. 10.1186/s12912-024-01841-z. 38515082 PMC10958847

[inr70232-bib-0002] Ariño‐Mateo, E. , R. Ramírez‐Vielma , M. Arriagada‐Venegas , G. Nazar‐Carter , and D. Pérez‐Jorge . 2022. “Validation of the Organizational Dehumanization Scale in Spanish‐Speaking Contexts.” International Journal of Environmental Research and Public Health 19, no. 8: 4805. 10.3390/ijerph19084805. 35457680 PMC9032923

[inr70232-bib-0003] Baer, M. , and M. Frese . 2003. “Innovation Is Not Enough: Climates for Initiative and Psychological Safety, Process Innovations, and Firm Performance.” Journal of Organizational Behavior 24, no. 1: 45–68. 10.1002/job.179.

[inr70232-bib-0004] Bakker, A. B. , E. Demerouti , A. Sanz‐Vergel , and A. Rodríguez‐Muñoz . 2023. “Job Demands‐Resources Theory: New Developments Over the Last Decade.” Revista de Psicologia del Trabajo y de las Organizaciones 39, no. 3: 157–167. 10.5093/JWOP2023A17.

[inr70232-bib-0005] Bowlby, J. 1982. “Attachment and Loss: Retrospect and Prospect.” American Journal of Orthopsychiatry 52, no. 4: 664–678. 10.1111/j.1939-0025.1982.tb01456.x. 7148988

[inr70232-bib-0006] Caesens, G. , F. Stinglhamber , S. Demoulin , and M. De Wilde . 2017. “Perceived Organizational Support and Employees' Well‐Being: The Mediating Role of Organizational Dehumanization.” European Journal of Work and Organizational Psychology 26: 527–540. 10.1080/1359432X.2017.1319817.

[inr70232-bib-0007] Cimon, L. , M. Gauthier , and M. Beaulieu . 2024. “Organizational Dehumanization of Nurses in Healthcare Systems: A Scoping Review.” The Annals of Family Medicine 22, no. Suppl 1: 6460. 10.1370/afm.22.s1.6460.

[inr70232-bib-0008] de Zulueta, P. C. 2015. “Developing Compassionate Leadership in Health Care: An Integrative Review.” Journal of Healthcare Leadership 8: 1–10. 10.2147/JHL.S93724. 29355200 PMC5741000

[inr70232-bib-0009] Demerouti, E. , A. B. Bakker , F. Nachreiner , and W. B. Schaufeli . 2001. “The Job Demands‐Resources Model of Burnout.” Journal of Applied Psychology 86, no. 3: 499–512. 10.1037/0021-9010.86.3.499. 11419809

[inr70232-bib-0010] DeVellis, R. F. , and C. T. Thorpe . 2021. Scale Development: Theory and Applications. Sage Publications.

[inr70232-bib-0011] Edmondson, A. 1999. “Psychological Safety and Learning Behavior in Work Teams.” Administrative Science Quarterly 44, no. 2: 350–383. 10.2307/2666999.

[inr70232-bib-0012] Edmondson, A. C. , and Z. Lei . 2014. “Psychological Safety: The History, Renaissance, and Future of an Interpersonal Construct.” Annual Review of Organizational Psychology and Organizational Behavior 1: 23–43. 10.1146/annurev-orgpsych-031413-091305.

[inr70232-bib-0013] Epp, K. 2012. “Burnout in Critical Care Nurses: A Literature Review.” Dynamics 23, no. 4: 25–31. https://pubmed.ncbi.nlm.nih.gov/23342935/. 23342935

[inr70232-bib-0014] Fajardo Castro, L. V. , A. M. Manrique Torres , I. M. Camargo Escobar , and M. C. Aguilar Bustamante . 2024. “Seguridad Psicológica en el Trabajo en población colombiana: Adaptación Lingüística y Validación de dos escalas [Psychological safety at work in the Colombian population: Linguistic adaptation and validation of two scales].” Revista Iberoamericana de Diagnóstico y Evaluación—e Avaliação Psicológica 74, no. 4: 151. 10.21865/RIDEP74.4.10.

[inr70232-bib-0015] Grailey, K. , C. Leon‐Villapalos , E. Murray , and S. Brett . 2021. “Exploring the Factors That Promote or Diminish a Psychologically Safe Environment: A Qualitative Interview Study With Critical Care Staff.” BMJ Open 11, no. 8: e046699. 10.1136/bmjopen-2020-046699. PMC8340293 34348949

[inr70232-bib-0016] Guo, Y.‐F. , J.‐Y. Fan , L. Lam , et al. 2022. “Associations Between Perceived Overqualification, Transformational Leadership and Burnout in Nurses From Intensive Care Units: A Multicentre Survey.” Journal of Nursing Management 30, no. 7: 3330–3339. 10.1111/jonm.13774. 36042016

[inr70232-bib-0017] Hair, J. F. , W. C. Black , B. J. Babin , and R. E. Anderson . 2019. Multivariate Data Analysis. Cengage.

[inr70232-bib-0018] Hair, J. , G. T. M. Hult , C. Ringle , and M. Sarstedt . 2022. A Primer on Partial Least Squares Structural Equation Modeling (PLS‐SEM). 3rd ed. Sage Publishing.

[inr70232-bib-0019] Harman, H. H. 1976. Modern Factor Analysis. University of Chicago Press.

[inr70232-bib-0020] Haruna, Y. , M. Shiromaru , and M. Sumikawa . 2023. “Factors Related to Intensive Care Unit Nurses' work Engagement: A Web‐Based Survey.” Nursing & Health Sciences 25, no. 3: 445–455. 10.1111/nhs.13041. 37562774

[inr70232-bib-0021] Haslam, N. 2006. “Dehumanization: An Integrative Review.” Personality and Social Psychology Review 10, no. 3: 252–264. 10.1207/s15327957pspr1003_4. 16859440

[inr70232-bib-0022] Iacopo, C. , N. Chiara , D. S. Matteo , Z. Lucia , C. Laura , and C. Guglielmo . 2024. “Dehumanization in Intensive Care Units and Standard Wards: a Survey for a Comparative Cross‐Sectional Study.” Applied Nursing Research: ANR 75: 151774. 10.1016/j.apnr.2024.151774. 38490798

[inr70232-bib-0023] Instituto Nacional de Estadística. INE . 2024. *Enfermeros Colegiados por Tipo de Especialidad, Año y Sexo* [Registered nurses by specialty type, year, and sex]. Spanish National Statistics Institute (INE). https://www.ine.es/jaxi/Datos.htm?tpx=49002.

[inr70232-bib-0024] Intriago Moreira, K. L. , M. F. Farfán López , and V. S. Rivas Hidalgo . 2025. “Riesgos Psicosociales en el Personal de Enfermería.” REFCalE: Revista Electrónica Formación y Calidad Educativa 13, no. 1: 57–68. 10.56124/refcale.v13i1.004.

[inr70232-bib-0025] Jamovi Project . 2024. *Jamovi* (Version 2.6) [Computer software]. https://www.jamovi.org.

[inr70232-bib-0026] Kappes, M. , P. Delgado‐Hito , V. R. Contreras , and M. Romero‐García . 2023. “Prevalence of the Second Victim Phenomenon Among Intensive Care Unit Nurses and the Support Provided by Their Organizations.” Nursing in Critical Care 28, no. 6: 1022–1030. 10.1111/nicc.12967. 37614030

[inr70232-bib-0027] Kock, N. , and P. Hadaya . 2018. “Minimum Sample Size Estimation in PLS‐SEM: The Inverse Square Root and Gamma‐Exponential Methods.” Information Systems Journal 28: 227–261. 10.1111/isj.12131.

[inr70232-bib-0028] Konečná, L. , E. Lisá , and V. Čiriková . 2026. “The Role of Leadership in Shaping Psychological Safety: A Qualitative Study From Slovakia.” Scientific Reports 16, no. 1: 7249. 10.1038/s41598-026-38706-1. 41639225 PMC12923528

[inr70232-bib-0029] Laguia, A. , S. Edú‐Valsania , M. Navas‐Jiménez C. del , F. Molero , and J. Moriano . 2025. “Liderazgo de Base Segura: Teoría e Investigación [Secure base leadership: Theory and research].” Anuario de Psicología/The UB Journal of Psychology 55, no. 2: 27–39. 10.1344/anpsic2025.55.2.3.

[inr70232-bib-0030] Lobato, P. , J. A. Moriano , A. Laguía , F. Molero , and M. Mikulincer . 2024. “Security Providing Leadership and Work Stress in Spanish Air Force.” Military Psychology 36, no. 5: 504–515. 10.1080/08995605.2023.2218785. 37256575 PMC11407392

[inr70232-bib-0031] Maslach, C. , and S. E. Jackson . 1981. “The Measurement of Experienced Burnout.” Journal of Organizational Behavior 2, no. 2: 99–113. 10.1002/JOB.4030020205.

[inr70232-bib-0032] Maslach, C. , and M. P. Leiter . 2008. The Truth About Burnout: How Organizations Cause Personal Stress and What to Do About It. John Wiley & Sons.

[inr70232-bib-0033] Molero, F. , M. Mikulincer , P. R. Shaver , A. Laguía , and J. A. Moriano . 2019. “The Development and Validation of the Leader as Security Provider Scale.” Revista de Psicología del Trabajo y de las Organizaciones 35, no. 3: 183–193. 10.5093/jwop2019a20.

[inr70232-bib-0034] Montenegro Méndez, S. , A. Laguía González , and J. A. Moriano León . 2025. “Is Leadership a Resource? A Systematic Review of Its Role in Burnout and Engagement Among Nurses Within the JD‐R Model.” Journal of Nursing Management 8853148. 10.1155/jonm/8853148. 41355851 PMC12677997

[inr70232-bib-0035] Moriano, J. A. , F. Molero , A. Laguía , M. Mikulincer , and P. R. Shaver . 2021. “Security Providing Leadership: A Job Resource to Prevent Employees' Burnout.” International Journal of Environmental Research and Public Health 18, no. 23: 12551. 10.3390/ijerph182312551. 34886276 PMC8657187

[inr70232-bib-0036] Moriano León, J. A. 2025. *Liderazgo de Base Segura y Gestión de Equipos* [Secure Base Leadership and Team Management]. Sanz y Torres.

[inr70232-bib-0037] Movaghar, M. , M. Alizadeh Sani , and Z. Andevari Sorek . 2024. “The Effect of Secure Base Leadership on Nursing Ethical Behaviors: Determining the Role of Individual Resilience Moderator. (Case study: Governmental hospitals in Mazandaran province).” Journal of Executive Management 15, no. 30: 335–359. 10.22080/jem.2023.23923.3772.

[inr70232-bib-0038] Navas‐Jiménez, M. C. , A. Laguía , P. Recio , et al. 2025. “The Buffering Effect of Secure Base Leadership on the Relationship Between Emotional Demands and Burnout: A Multilevel Study Among Military Officer Cadets.” Acta Psychologica 255: 104971. 10.1016/j.actpsy.2025.104971. 40194486

[inr70232-bib-0039] Navas‐Jiménez, M. C. , A. Laguía , P. Recio , et al. 2024. “Secure Base Leadership in Military Training: Enhancing Organizational Identification and Resilience Through Work Engagement.” Frontiers in Psychology 15: 1401574. 10.3389/fpsyg.2024.1401574. 39776970 PMC11703663

[inr70232-bib-0040] Padilha, K. G. , R. L. Barbosa , R. Andolhe , et al. 2017. “Carga de Trabalho de Enfermagem, Estresse/Burnout, Satisfação e Incidentes em unidade de terapia intensiva de trauma.” Texto & Contexto—Enfermagem 26, no. 3: e1720016. 10.1590/0104-07072017001720016.

[inr70232-bib-0041] Perveen, K. 2022. “Role of Nurses in Intensive Care Unit.” NURSEARCHER (Journal of Nursing & Midwifery Sciences) 2, no. 1: 01. 10.54393/nrs.v2i01.22.

[inr70232-bib-0042] Podsakoff, P. M. , S. B. MacKenzie , J.‐Y. Lee , and N. P. Podsakoff . 2003. “Common Method Biases in Behavioral Research: A Critical Review of the Literature and Recommended Remedies.” Journal of Applied Psychology 88, no. 5: 879–903. 10.1037/0021-9010.88.5.879. 14516251

[inr70232-bib-0043] Quesada‐Puga, C. , F. J. Izquierdo‐Espin , M. J. Membrive‐Jiménez , et al. 2024. “Job Satisfaction and Burnout Syndrome Among Intensive‐care Unit Nurses: A Systematic Review and Meta‐Analysis.” Intensive and Critical Care Nursing 82: 103660. 10.1016/j.iccn.2024.103660. 38394983

[inr70232-bib-0044] Ramírez‐Elvira, S. , J. L. Romero‐Béjar , N. Suleiman‐Martos , et al. 2021. “Prevalence, Risk Factors and Burnout Levels in Intensive Care Unit Nurses: A Systematic Review and Meta‐analysis.” International Journal of Environmental Research and Public Health 18, no. 21: 11432. 10.3390/ijerph182111432. 34769948 PMC8583312

[inr70232-bib-0045] Ringle, C. M. , S. Wende , and J. M. Becker . 2022. SmartPLS 4 [Computer software]. SmartPLS GmbH. http://www.smartpls.com.

[inr70232-bib-0046] Saravanabavan, L. , and V. Poongavanam . 2023. “Perceived Organisation Support Would Buffer the Impact of Work Frustration on Burn‐Out Among Intensive Care Unit Nurses.” Evidence Based Nursing 26, no. 4: 142. 10.1136/ebnurs-2023-103716. 37349089

[inr70232-bib-0047] Schaufeli, W. B. , S. Desart , and H. De Witte . 2020. “Burnout Assessment Tool (BAT): Development, Validity, and Reliability.” International Journal of Environmental Research and Public Health 17, no. 24: 9495. 10.3390/ijerph17249495. 33352940 PMC7766078

[inr70232-bib-0048] Schaufeli, W. B. , M. Salanova , V. González‐romá , and A. B. Bakker . 2002. “The Measurement of Engagement and Burnout: A Two‐Sample Confirmatory Factor Analytic Approach.” Journal of Happiness Studies 3, no. 1: 71–92. 10.1023/A:1015630930326/METRICS.

[inr70232-bib-0049] Schaufeli, W. B. , A. Shimazu , J. Hakanen , M. Salanova , and H. De Witte . 2019. “An Ultra‐Short Measure for Work Engagement.” European Journal of Psychological Assessment 35, no. 4: 577–591. 10.1027/1015-5759/a000430.

[inr70232-bib-0050] Schwarzkopf, D. , J. Felfe , C. Hartog , and F. Bloos . 2015. “Making It Safe to Speak up About Futile Care: A Multiperspective Survey on Leadership, Psychological Safety and Perceived Futile Care in the ICU.” Critical Care 19, no. 1: P567. 10.1186/cc14647.

[inr70232-bib-0051] Soriano, A. , H. Warsicka , and J. M. Peiró . 2025. “The Burnout Assessment Tool (BAT): Validation in the Spanish Population.” Work 81, no. 2: 2791–2800. 10.1177/10519815251320276. 40421559

[inr70232-bib-0052] Wang, T. , A. C. M. Abrantes , and Y. Liu . 2023. “Intensive Care Unit Nurses' Burnout, Organizational Commitment, Turnover Intention and Hospital Workplace Violence: A Cross‐Sectional Study.” Nursing Open 10, no. 2: 1102–1115. 10.1002/nop2.1378. 36126210 PMC9834521

[inr70232-bib-0053] Yin, J. , L. Zhao , N. Zhang , and H. Xia . 2024. “Understanding the Interplay of Compassion Fatigue and Moral Resilience on Moral Distress in ICU Nurses: A Cross‐sectional Study.” Frontiers in Public Health 12: 1402532. 10.3389/fpubh.2024.1402532. 39529716 PMC11550935

[inr70232-bib-0054] Yousif, S. Y. , and S. Al‐Fayyadh . 2025. “Burnout Among Nurses Practicing in Critical Care Units: Predicting the Contributing Factors.” Journal of Education and Health Promotion 14: 38. 10.4103/jehp.jehp_926_24. 40104345 PMC11918301

[inr70232-bib-0055] Zhang, L. , L. Han , X. Liang . et al., 2025. “The Relationship Between Transformational Leadership and Work Engagement Among Intensive Care Unit Nurses: The Mediating Function of Organizational Climate.” BMC Nursing 24, no. 1: 398. 10.1186/s12912-025-03057-1. 40211298 PMC11984227

